# Population genomics in two cave-obligate invertebrates confirms extremely limited dispersal between caves

**DOI:** 10.1038/s41598-020-74508-9

**Published:** 2020-10-16

**Authors:** Andras Balogh, Lam Ngo, Kirk S. Zigler, Groves Dixon

**Affiliations:** 1 Department of Integrative Biology, University of Texas, PAT Building Room 427, 2401 Speedway, Austin, TX USA; 2 Department of Biology, University of the South, Sewanee, TN USA

**Keywords:** Biodiversity, Biogeography, Molecular ecology, Evolutionary genetics, Population genetics

## Abstract

Caves offer selective pressures that are distinct from the surface. Organisms that have evolved to exist under these pressures typically exhibit a suite of convergent characteristics, including a loss or reduction of eyes and pigmentation. As a result, cave-obligate taxa, termed troglobionts, are no longer viable on the surface. This circumstance has led to an understanding of highly constrained dispersal capabilities, and the prediction that, in the absence of subterranean connections, extreme genetic divergence between cave populations. An effective test of this model would involve (1) common troglobionts from (2) nearby caves in a cave-dense region, (3) good sample sizes per cave, (4) multiple taxa, and (5) genome-wide characterization. With these criteria in mind, we used RAD-seq to genotype an average of ten individuals of the troglobiotic spider *Nesticus barri* and the troglobiotic beetle *Ptomaphagus hatchi*, each from four closely located caves (ranging from 3 to 13 km apart) in the cave-rich southern Cumberland Plateau of Tennessee, USA. Consistent with the hypothesis of highly restricted dispersal, we find that populations from separate caves are indeed highly genetically isolated. Our results support the idea of caves as natural laboratories for the study of parallel evolutionary processes.

## Introduction

Caves are unique habitats with environmental conditions fundamentally distinct from the surface. The most conspicuous of these is the complete absence of light, precluding the use of visual cues for hunting, foraging, locating mates, and evading predators^1^. Moreover, as photosynthesis is not possible, cave communities depend nearly entirely on trophic input from the surface. Caves are also more stable in temperature and humidity than surface habitats^2^. As a result, adaptation to life underground typically involves extensive evolutionary change^3^.

Some organisms have evolved under these conditions to the extent that they are never found outside of caves. These organisms, termed troglobionts^2^, often bear a suite of distinctive characteristics, including loss or reduction of eyes, pigment loss, elongated appendages, improved non-visual sensory mechanisms, reduced metabolic rates, longer lifespans, and lower rates of reproduction^2–4^. These features represent widely repeated cases of convergent evolution, that can be shaped even by fine-scaled niche partitioning within subterranean habitats^5^. Many of these phenotypes have obvious tradeoffs for fitness on the surface. For instance, pigment loss, selectively neutral or possibly even advantageous in the cave environment^6^, will decrease crypsis on the surface, a trait known to undergo particularly strong purifying selection^7^. While most studies the selective pressures on crypsis come from surface-dwelling species^8^, the importance of camouflage among other arthropods is suggestive that similar pressures would exist for troglobionts traversing surface terrain. Intolerance to variation in temperature and humidity may also preclude surface viability^2^. Hence, the surface is a hostile environment for troglobionts. With this idea in mind, Culver and Pipan^2^ pointed out that caves are like islands in a sea of surface habitat.

Previous studies have shown that troglobiont migration is indeed highly limited. For instance, at the species level, endemism is less the exception than the rule^9,10^. This is especially true in the eastern United States, where up to 45% of troglobionts are single-cave endemics^11^. These exceptional rates of endemism are consistent with restricted gene flow and frequent speciation. Population genetic studies lend further support. Examining COI in the troglobiotic spider *Nesticus barri*, Snowman et al*.*^12^ found extensive haplotype divergence and limited sharing of haplotypes between caves, indicating that migration was minimal to nonexistent over distances greater than 15 km. Another study, examining COI in several troglobionts, including *N. barri* and the beetle *Ptomaphagus hatchi*, provided similar findings^13^, indicating that restricted migration is likely general to terrestrial troglobionts.

**Figure 1:**
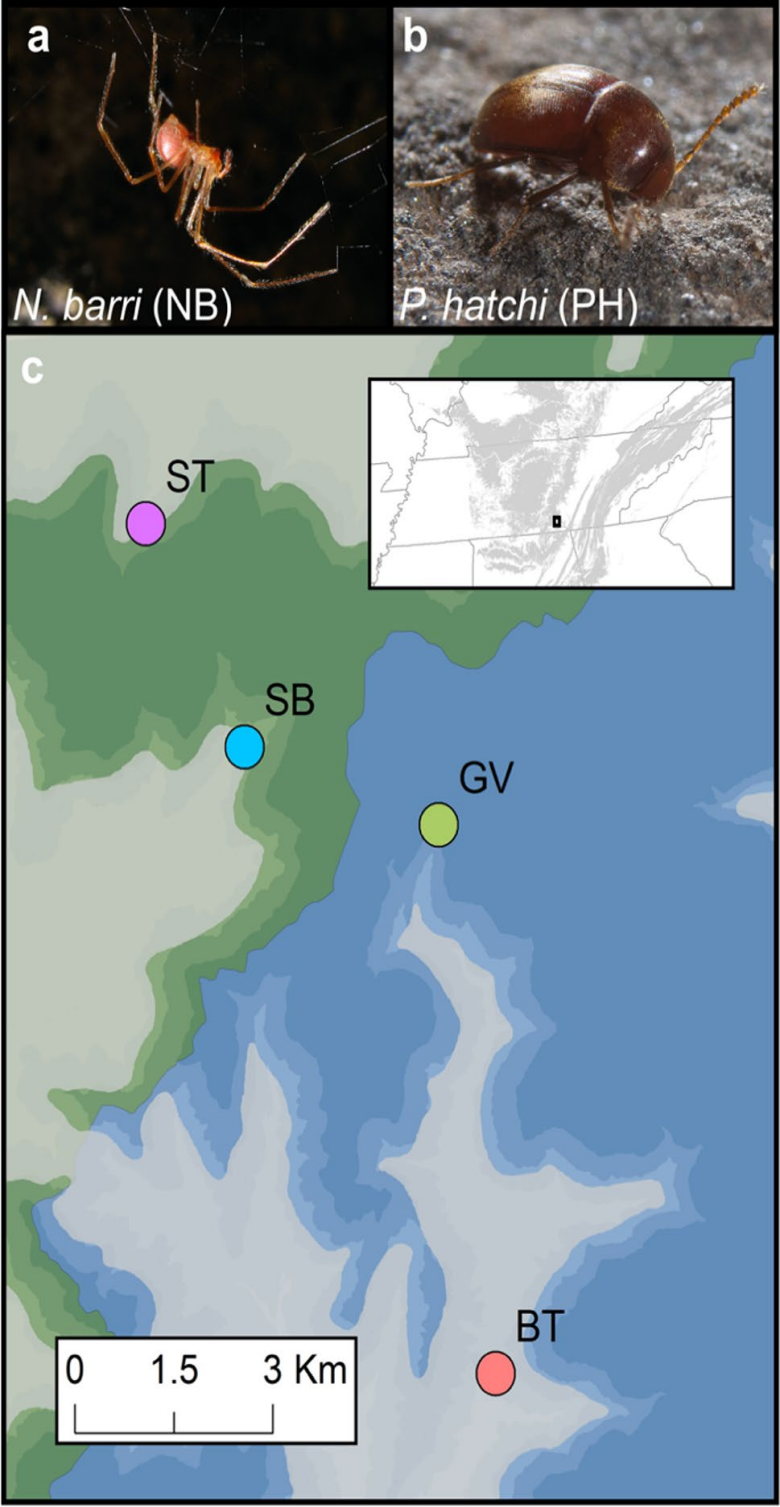
Study location and abbreviations. (**a**) *Nesticus barri* (NB), (**b**) *Ptomaphagus hatchi* (PH), (**c**) Map of sample cave locations: *BT* Buggytop, *GV* Grapevine, *SB* Sewanee Blowhole, *ST* Solomon’s Temple. The Upper Elk River watershed is indicated by green coloration and the Guntersville Lake watershed is indicated by blue coloration. Shading intensity indicates elevation with higher elevations in darker tones. The map was created using ArcGIS 10.6 (desktop.arcgis.com). Photos by (**a**) Alan Cressler and (**b**) Michael Slay.

Here, we evaluate the hypothesis that dispersal of terrestrial troglobiont invertebrates is extremely limited between caves. To emphasize the severity of isolation, we sampled individuals from caves located close together on the southern Cumberland Plateau, one of the most biodiverse karst areas in the United States^9,10,14^ (Fig. 1; Table 1). The southern Cumberland Plateau is also one of the most cave-rich regions in North America, with more than 4000 caves known from a six-county area in southern Tennessee and northeast Alabama^15^. To ensure the generality of the hypothesis, we focused on two species with distinct natural histories: the spider *N. barri* and the beetle *P. hatchi*. *N. barri* is part of a complex of 28 species found across the southeastern United States^16^. Their tendency to live in dark, moist habitats has led to numerous instances of cave habitation, with roughly one third of species in the group either troglophiles (frequent cave dwellers that are also found on the surface) or troglobionts^17^. *N. barri* demonstrates typical troglomorphic features, lacking eyes and with reduced pigment, although it still possesses reproductive seasonality^18^. The genus *Ptomaphagus* includes about 60 species in North America, again with roughly one third either troglophiles or troglobionts^4^. Diversification of the genus throughout the southern Cumberland Plateau is thought to have occurred through progressive vicariance, as the Cumberland Plateau eroded over the last 6 million years^19^. Like other troglobiotic *Ptomaphagus*, *P. hatchi* has greatly reduced eyes and is wingless. On the southern Cumberland Plateau, *N. barri* and *P. hatchi* have largely overlapping ranges, with each species known from dozens of caves^12,19^, and both species are common in the caves they inhabit. Finally, where previous studies made use of one, or at most a handful loci, we take a genome-wide approach using 2bRAD^20^. This method has the advantage of interrogating thousands of loci across the genome, allowing for more confident estimates of population divergence and neutral diversity^21,22^. This is the first study to investigate these species at the genomic scale.

**Table 1:** Cave and sampling information.

Cave	Abbreviation	Survey #	Length (m)	Watershed	Sample size	
					*N. barri*	*P. hatchi*
Solomon’s Temple Cave	ST	FR26	370	Upper Elk River	12	6
Sewanee Blowhole	SB	FR91	1219	Upper Elk River	8	7
Grapevine Cave	GV	FR423	490	Guntersville Lake	10	12
Buggytop Cave	BT	FR16	3142	Guntersville Lake	16	7

With this approach, we test several predictions of the hypothesis of extremely limited dispersal between caves. First, each cave should harbor a genetically distinct population. Individuals from any cave should be most similar to others from the same cave. Hence, unsupervised methods such as hierarchical clustering, and principal component analysis should sort samples based on their cave of origin. Second, when modeled as mixtures of ancestral populations, individuals should demonstrate limited admixture between caves. Third, we should observe extensive genetic differentiation between caves. We test this third prediction using pairwise estimates of F_ST_ and d_XY_. An important corollary of our hypothesis is that intermediate subterranean connectivity, via habitats such as horizontal fissures^5^ or Mesovoid Shallow Substratum (MSS)^23,24^ is also highly limited or nonexistent.

## Materials and methods

### Sampling

We collected specimens from four caves on the edge of the southern Cumberland Plateau in Franklin County, Tennessee (Table 1). The caves were chosen based on proximity, location, and previous knowledge of the presence of *N. barri* and *P. hatchi*^13,25^. Distances between the caves ranged from 3 to 13 km (Fig. 1). The caves are distributed across two adjacent watersheds, with Solomon’s Temple (ST) and Sewanee Blowhole (SB) in the Upper Elk River watershed that drains to the north and west of the study area, whereas Grapevine Cave (GV) and Buggytop Cave (BT) are in the Guntersville Lake watershed that drains the study area to the south (Fig. 1; Table 1). Three of the caves (ST, GV, and BT) have maps on record with the Tennessee Cave Survey, so for those caves, we had a precise measure of the total human-accessible cave passage. The fourth cave (SB) has not been mapped, so we relied on the estimated length of the cave as noted in the Tennessee Cave Survey records.

Sampling was conducted between 21 September and 25 October 2018. We collected *Ptomaphagus* and *Nesticus* by hand during visual encounter surveys as two researchers moved through the cave. Sampling was opportunistic, rather than random, but was dispersed throughout the cave. An initial survey of Buggytop Cave yielded only a few *Ptomaphagus*, so food baits (tuna) were placed in the cave for 24 h and live specimens were subsequently collected at the baits. Sample size per cave ranged from 6 to 16 individuals per species (Table 1). Specimens were placed into 100% EtOH in the field and subsequently stored at − 20 °C. Sampling was permitted by the Tennessee Wildlife Resources Agency (Permit #1385) and the Tennessee Department of Environment and Conservation (Permit #2013-026).

### Library preparation

Most of the DNA extractions were performed using the entire body of the individual. If a particular sample seemed large enough (mainly applied to *Nesticus barri*) the legs were saved while the cephalothorax and abdomen were used. QIAGEN’s DNeasy Blood & Tissue Kit (Cat. No. 69504 or 69506) was used following the kit’s protocol with the exception of using 50 µl Buffer AE for elution. Concentrations of each DNA isolation were initially checked by nanodrop and confirmed with the Quant-IT Picogreen DS DNA assay (Life Technologies cat. no. P7589). The 2b-RAD library preparation was carried out as described previously^20,26–28^. Briefly, DNA isolations were normalized to ~ 12.5 ng/µl. Samples with concentrations lower than 12.5 ng/µl DNA (~ 30 samples), were fully dehydrated in a vacuum centrifuge and resuspended to a target concentration of ~ 12.5 ng/µl. Digestion reactions had concentrations of 1 × NEB buffer #3 and 10 µM SAM mixed with 1 total U of BcgI restriction enzyme and 50 ng genomic DNA in a total volume of 6 µl. Digests were incubated at 37 °C for 1 h followed by 20 min at 65 °C for heat inactivation. Ligation reactions had concentrations of 1 × T4 ligase buffer, and 0.25 µM each adapter with 400 total U of T4 DNA ligase and 6 µl of digested DNA in a total volume of 20 µl. Ligation reactions were incubated at 4 °C overnight, followed by 20 min at 65 °C for heat inactivation. Inclusion of internal barcodes in the i7 adapter allowed for pooling sets of samples at this point. Amplification and additional barcoding reactions were performed on these pools. These reactions had concentrations of 312 µM each dNTP, 0.2 µM each of p5 and p7, 0.15 µM appropriate TruSeq-Un primer, 0.15 µM appropriate primer, 1 × Titanium Taq buffer, and 1 × Taq polymerase mixed with 4 µl of pooled ligation in a total volume of 20 µl. Thermocycler conditions were 70 °C for 30 s, followed by 14 cycles of 95 °C for 20 s, 65 °C for 3 min, and 72 °C for 30 s. The final library was pooled into a single tube and sequenced at the University of Texas at Austin’s Genome Sequencing and Analysis Facility on a single lane on the Illumina Hiseq 2500 platform.

### Data processing

Raw reads were trimmed and demultiplexed based on internal ligation barcodes using custom Perl scripts^28^. Reads were quality filtered using fastq_quality_filter from the Fastx Toolkit (https://hannonlab.cshl.edu/fastx_toolkit/). Generation of de novo loci was performed using cd-hit^29^ and custom Perl scripts as described previously^20,26–28^. These tags were assembled into 30 equally sized pseudo-chromosomes for mapping. Re-mapping of reads to these de novo loci was done with bowtie2^30^. Sorting and indexing of bam files in preparation for genotyping were done with samtools^31^.

### Genotype analyses

We analyzed the genotype data in two ways. The first method depended on hard genotype calls, in which the genotype of each individual at each site is either called exactly or filtered to missing data based on arbitrary cutoffs. While simpler to implement, these hard genotype calls can introduce biases, because they can fail to capture statistical uncertainty inherent to individual genotypes from Next-Generation Sequencing data^32^. A second, alternative approach is to estimate sample allele frequency spectra directly from base calls and quality metrics in the alignment data, allowing for population genetic inferences without making individual genotype calls^32^. We processed hard genotype calls primarily using VCFtools^33^. Estimation of genotype likelihoods, allele frequency spectra, and additional population genetic inferences were implemented using Angsd^34–36^. Throughout the manuscript we emphasize the results produced using Angsd, using analyses from hard genotype calls primarily for corroboration. All steps used for both sets of analyses, along with scripts for statistical analysis and figure generation are included in the git repository^37^.

Hard genotype calls were made as described previously^38^ using mpileup and bcftools^39^. Genotype calls with depth lower than 2, as well as indels, singletons, sites with more than two alleles, or less than 75% of samples genotyped were removed using VCFtools^33^. Sites with excess heterozygosity (*p* value < 0.1) (likely paralogs) were removed based on the—hardy output from VCFtools.

For the analyses performed with Angsd, quality filtering included a minimum mapping score of 20 (− minMapQ 20), a minimum quality score of 32 (− minQ 32), and minimum representation among samples > 80% (− minInd). Filters based on *p* values, including the strand bias *p* value (− sb_pvalue)^40^, the hetbias *p* value (*−* hetbias_pval)^41^, and SNP *p* value (− snp_pval), were set to 0.05.

### Summarizing genetic variation

To summarize genetic variation, we used Angsd to calculate pairwise differences between samples using the − IBS 1 option and a minimum minor allele frequency of 1% (− minMaf). Here pairwise distance between samples *i* and *j* (*d*_*ij*_) is calculated as:

$${d}_{ij}=\frac{{\sum }_{m}^{M}1-{I}_{{b}_{j}}\left({b}_{i}\right)}{M}$$

where *M* is the total number of sites with at least 1 read from each individual, and 1 − *I*_*bj*_(*b*_*i*_) is the indicator function which is equal to one when the two individuals have the same base and zero otherwise^34^. This distance matrix was used for hierarchical clustering and multidimensional scaling. We determined the optimal number of mixture model components for this distance matrix based on BIC using the Mclust package^42^. Admixture analysis was performed on genotype likelihoods output by Angsd using NGSadmix^43^.

### Genetic differentiation between populations based on SNP calls

We estimated F_ST_ for each pair of populations using Angsd^35,44^. This method computes the posterior expectation of genetic variance between populations (designated A), and total expected variance (designated B). These values (A and B) are closely related to the alpha and beta estimates described in Reynolds et al*.*^45^. The unweighted F_ST_ is computed as the mean of the per-site ratios of A and B and the weighted F_ST_ is computed as the ratio of the sum of As to the sum of Bs^34^. The unweighted and weighted F_ST_ values reported in Table 2 and Fig. 4 are these estimates computed from all sites in the dataset. Based on communications that unweighted F_ST_ values tend to be too noisy^36^ we focus on the weighted F_ST_ values.

**Table 2:** Estimates of pairwise genetic differentiation between caves.

Pair	Watershed	Species	Weighted	Unweighted	Weir	d_XY_
SB–ST	Same	*N. barri*	0.33	0.12	0.20	0.14
BT–GV	Same	*N. barri*	0.42	0.13	0.29	0.25
BT–ST	Different	*N. barri*	0.42	0.14	0.33	0.31
BT–SB	Different	*N. barri*	0.43	0.14	0.32	0.30
GV–ST	Different	*N. barri*	0.49	0.15	0.45	0.33
GV–SB	Different	*N. barri*	0.52	0.18	0.45	0.32
SB–ST	Same	*P. hatchi*	0.34	0.18	0.25	0.21
BT–GV	Same	*P. hatchi*	0.30	0.17	0.16	0.22
BT–ST	Different	*P. hatchi*	0.34	0.17	0.27	0.25
BT–SB	Different	*P. hatchi*	0.32	0.17	0.15	0.24
GV–ST	Different	*P. hatchi*	0.36	0.19	0.31	0.25
GV–SB	Different	*P. hatchi*	0.36	0.20	0.22	0.24

‘Weighted’ and ‘unweighted’ indicate the F_ST_ estimates computed with Angsd. ‘Weir’ indicates Weir and Cockerham’s F_ST_ estimate computed with VCFtools. ‘d_XY_’ indicates pairwise genetic distance computed from VCFtools allele frequencies using the equation given in the methods section.

Pairwise F_ST_ and d_XY_ were also calculated for each pair of caves based on hard genotypes. We used VCFtools to calculate pairwise Weir and Cockerham’s F_ST_^46^, and a custom R script to calculate d_XY_ for unphased data as:

$${d}_{XY}=\sum _{ij}{x}_{i}{y}_{j}{k}_{ij}$$

where *x*_*i*_ and *y*_*j*_ are the frequencies of the *i*th allele from population X and the *j*th allele from population Y respectively, and *k*_*ij*_ is 1 when *i* and *j* differ, and 0 if they are the same^47^. The Weir and Cockerham’s F_ST_ and the d_XY_ values reported in Table 2 and Fig. 4 are the averages of these statistics across all hard genotyped SNPs. We used our Weir and Cockerham’s F_ST_ values to estimate pairwise migration rates according to the following formula^47^:

$$E\left({F}_{ST}\right)=\frac{1}{1+4{N}_{e}m}.$$

### Nucleotide diversity

To calculate nucleotide diversity using genotype likelihoods, we first generated genotype likelihoods as described above. We then used the custom Python script HetMajorityProb.py^27,28^ to remove sites where the heterozygosity rate appeared higher than 50%, as these were likely paralogs spuriously lumped as single loci. We then estimated nucleotide diversity from the folded site frequency spectra using Angsd^35,48^. The value was then averaged across the 30 pseudochromsomes from the reference created during de novo locus generation described above. These averages are the values reported in Fig. 5.

We also calculated nucleotide diversity (π) from hard genotype calls. Here we first determined the allele frequencies for each species in each cave using VCFtools. We then calculated the expected heterozygosity (*h*) for each site as:

$$h=\frac{n}{n-1}\left(1-\sum {p}_{i}^{2}\right)$$

where *n* is the number of sequences, and *p*_*i*_ is the frequency of the *i*th allele at the site^47^. We then calculated π as the sum of expected heterozygosities across sites:

$$\pi =\sum_{j=1}^{S}{h}_{j}$$

where *S* is the number of segregating sites and *h*_*j*_ is the expected heterozygosity of the site^47^. We report this value per site by dividing by the total number of interrogated positions. Effective population size was estimated by dividing this per site value by four times the number of mutations per site per generation (4µ). The mutation rate estimate was based on *Drosophila*^49^.

## Results

### Sequencing

Sequencing produced 236 million raw reads. After demultiplexing and PCR duplicate removal, a total of 65.8 million reads remained. Mean fold coverage per sample tended to be higher for *P. hatchi* (1.2 ± se 0.08 million reads) than *N. barri* (0.462 ± se 0.04 million reads). De novo locus generation produced 130,061 loci for *N. barri* and 264,486 loci for *P. hatchi*. Filtering of hard genotype calls from mpileup produced 7620 biallelic SNPs for *N. barri* and 12,879 for *P. hatchi*. Filtering of genotype likelihoods produced with Angsd identified 13,629 SNPs for *N. barri* and 31,000 for *P. hatchi*. Final sample sizes for each species are given in Table 1.

### Delineating populations

**Figure 2:**
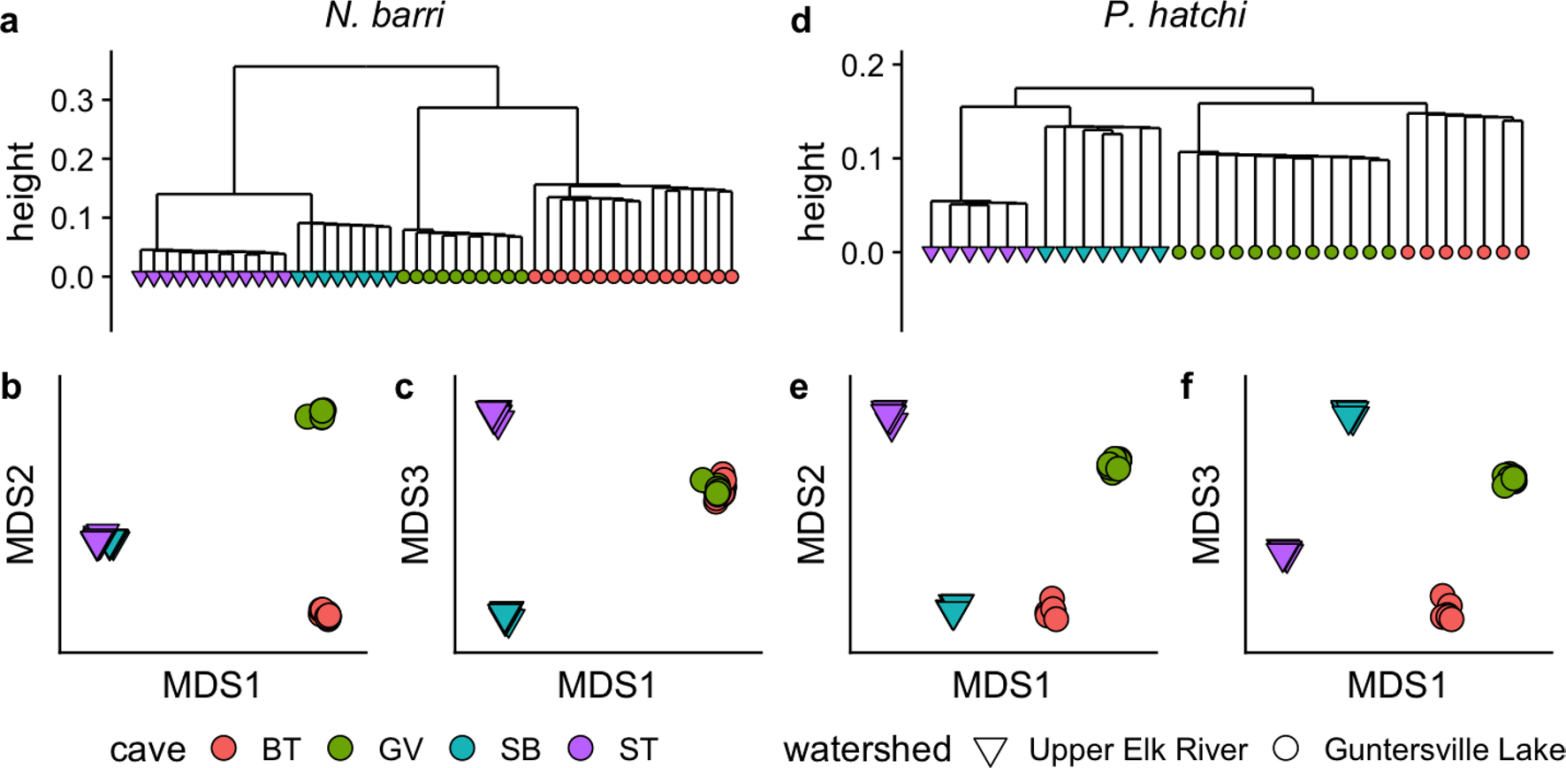
Sample clustering by cave. (**a**) Hierarchical clustering of *N. barri* samples based on pairwise distance. (**b**,**c**) Multidimensional scaling plots based on pairwise distance for *N. barri*. (**d**–**f**) Same plots for *P. hatchi*.

For both species, the four caves harbored genetically distinct populations. Hierarchical clustering based on pairwise differences separated individuals by cave (Fig. 2a,d). Overall topology of clustering was the same for both species, with caves from the same watershed clustering together (Figs. 1, 2a,d). Multidimensional scaling produced similar results, with clear clustering by cave along the first three axes for *N. barri* (Fig. 2b,c) and the first two for *P. hatchi* (Fig. 2e,f). Both analyses indicated that SB and ST caves were more similar for *N. barri* than *P. hatchi*.

**Figure 3:**
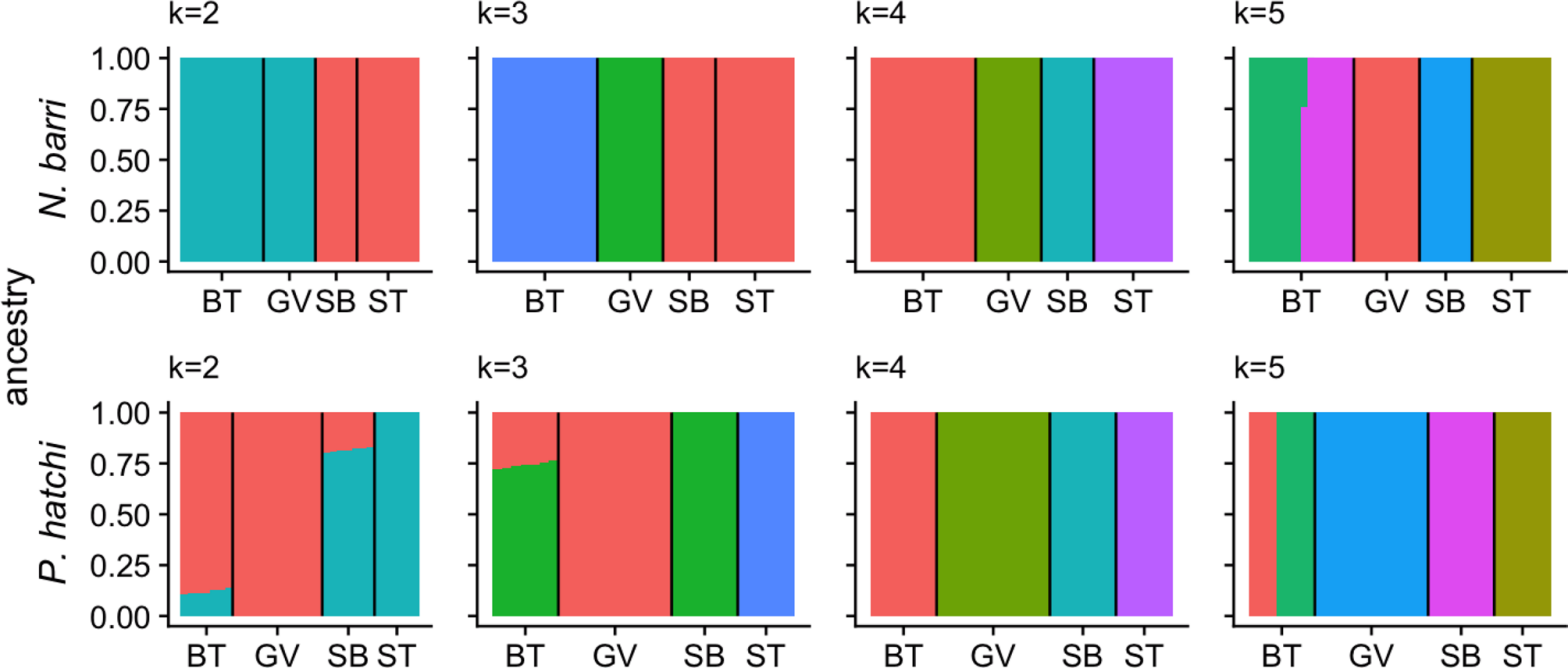
Admixture plots for varying numbers of ancestral populations (k = 2–5) for *N. barri* (top) and *P. hatchi* (bottom). For each panel, *k* indicates the number of ancestral populations assumed during the analysis and stacked bars represent estimates of proportional ancestry for each individual. Black vertical lines separate groups of individuals from different caves: (*BT* Buggytop, *GV* Grapevine, *SB* Sewanee Blowhole, *ST* Solomon’s Temple).

Admixture analysis further supported the caves as independent populations. For both species, the optimal number of mixture model components based on BIC was k = 4 or 5, with only marginal improvements in BIC for the 5th component (Fig. S2). When the number of ancestral populations (k) was set to four, ancestry estimates matched fully with source cave (Fig. 3). When k was set to 5, both species showed evidence of two groups within the largest cave, Buggy Top, rather than admixture between caves. Together, these analyses indicate that the four caves are indeed genetically distinct populations.

### Genetic differentiation between caves

**Figure 4:**
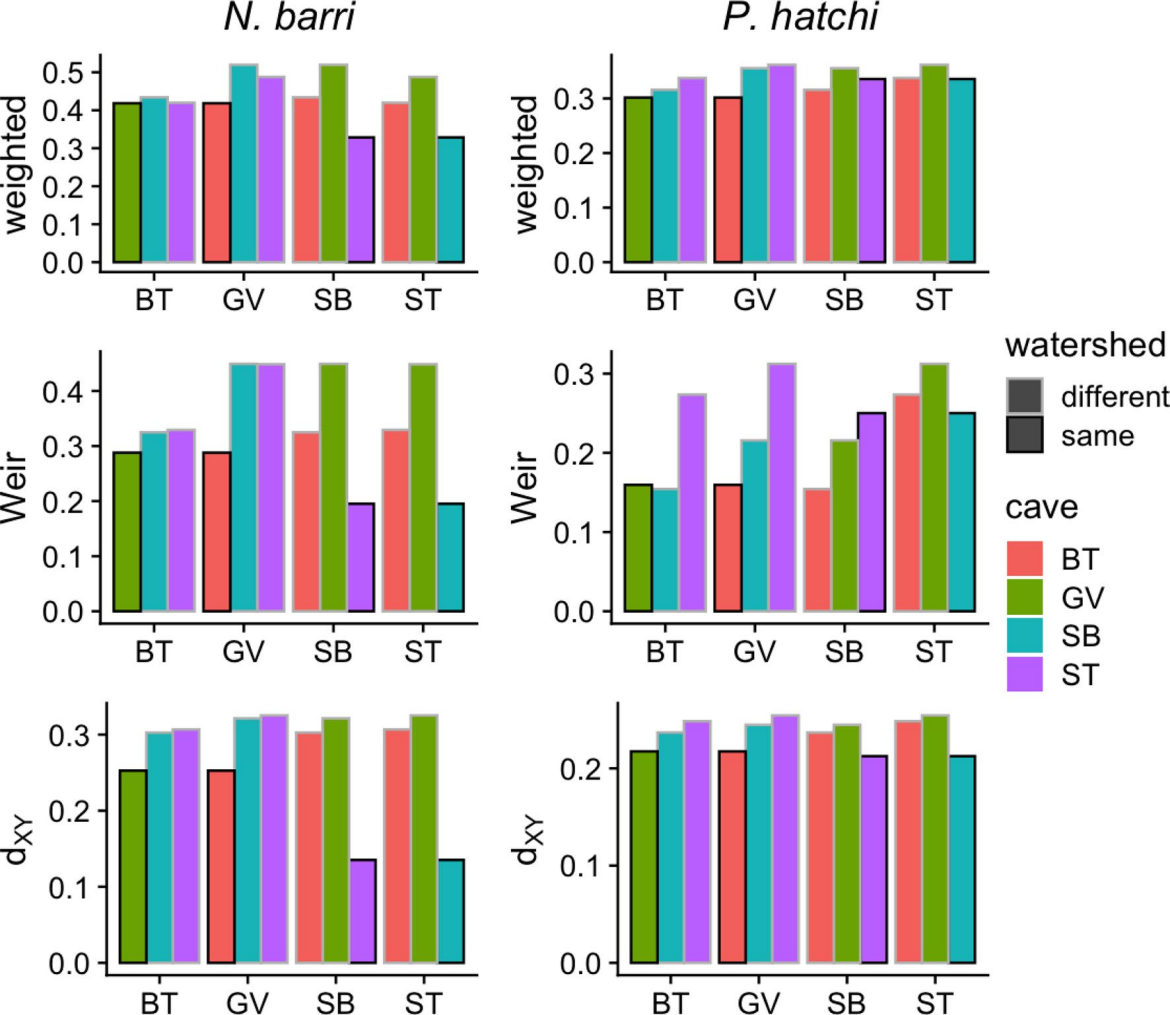
Pairwise estimates of genetic differentiation between caves. The first cave in each pair is indicated on the X axis. The second cave is indicated by the bar color. Whether the two caves are located in the same watershed is indicated by the bar outline color. The statistics are: weighted = weighted F_ST_ computed using Angsd; Weir = Weir and Cockerham’s^44^ F_ST_ averaged across all variant sites from hard genotype calls; d_XY_ = absolute genetic distance averaged across all variant sites from hard genotype calls.

Genetic differentiation between caves was high. Pairwise weighted F_ST_ (Angsd) ranged from 0.33 to 0.52 for *N. barri*, and 0.3 to 0.36 for *P. hatchi*. Unweighted F_ST_ (Angsd) and Weir and Cockerham’s F_ST_ estimated from hard genotype calls were lower, but still considerable, with a minimum value of 0.12 (Table 2; Fig. 4).

### Nucleotide diversity

**Figure 5:**
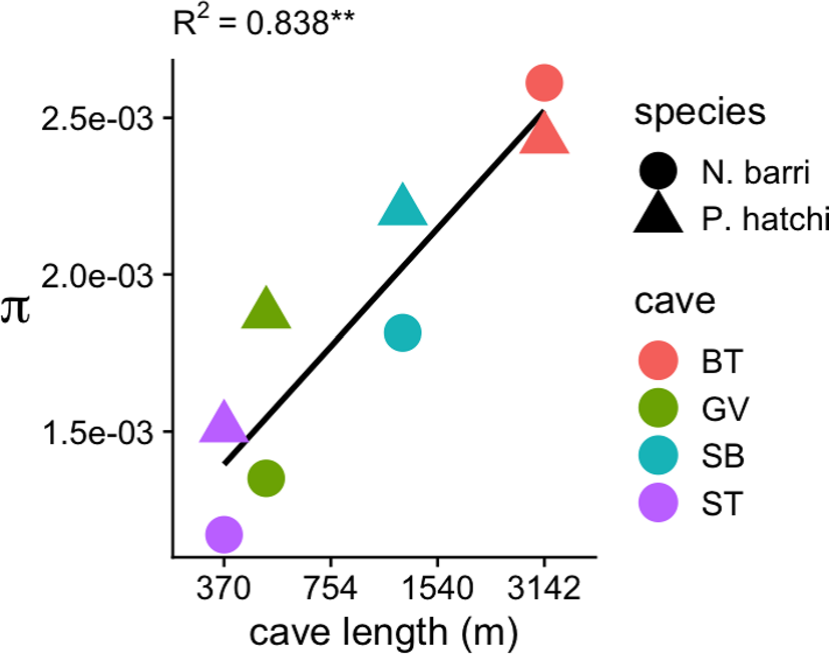
Relationship between per site nucleotide diversity (π) and cave length. The X-axis shows cave length on the log scale. The Y-axis shows the nucleotide diversities for the two species in each cave computed using Angsd. Point color indicates cave and point shape indicates species. Black line traces the linear regression for all points. R^2^ for the linear model for all points is given above the plot (*p* < 0.01).

In both species, estimates of nucleotide diversity based on genotype likelihoods indicated surprising levels of diversity that varied with cave length. For individual caves, per site nucleotide diversity (π) ranged from 1.17 × 10^–3^ to 2.43 × 10^–3^. For all but the longest cave (Buggytop Cave; BT), the beetle *P. hatchi* had higher nucleotide diversity than the spider *N. barri* (Table 3). While the sample size was small, we found that for both species, nucleotide diversity correlated positively with cave length (Fig. 5). In a multiple linear regression model of nucleotide diversity that included species, cave length, and cave name (R^2^ = 0.92), only cave length was significant when controlling for the other independent variables (*p* < 0.05). Assuming a mutation rate of 2.8 × 10^–9^ estimated for *Drosophila*^49^, the effective population sizes based on the π estimates for *P. hatchi* ranged from 1.4 × 10^5^ in Solomon’s Temple to 2.2 × 10^5^ in Buggytop, with a range of 1.0 × 10^5^ to 2.3 × 10^5^ for *N. barri* (Table 3). We note that the actual mutation rate for these species is unknown and that the estimate for Drosophila serves only as a rough approximation. Hence the resulting Ne estimates are highly uncertain, and differences between species could be influenced by differences in mutation rate as well as demography. Nucleotide diversity estimates based on hard genotype calls were proportionally similar, but on average eightfold lower than from genotype likelihoods (Table 3). This likely resulted from greater stringency during filtering of variant sites from the hard genotype calls. Estimates of π from hard genotype calls were similarly positively associated with cave length (Fig. S3). Based on our estimates of Weir and Cockerham’s F_ST_ between caves (Fig. 4; Table 2), we computed pairwise migration rates in terms of effective population size as M = 4Ne x m. This statistic ranged from 1.23 to 4.12 for *Nesticus* (mean = 2.19) and from 2.20 to 5.48 (mean = 3.71) for *Ptomaphagus*. Hence, the maximum migration rate *m* for any pair of caves was estimated to be less than 1.5 × 10^–5^. While our estimates of Ne are highly uncertain, this low value demonstrates that migration between caves is indeed highly restricted.

**Table 3:** Nucleotide diversity for each species and cave estimated using genotype likelihoods (Angsd) and from hard genotype calls (mpileup).

Species	Cave	Cave length (m)	π (Angsd)	Ne (Angsd)	π (mpileup)	Ne (mpileup)
*N. barri*	ST	370	1.2E−03	1.0E+05	1.6E−04	1.4E+04
*N. barri*	GV	490	1.4E−03	1.2E+E+05	2.0E−04	1.8E+04
*N. barri*	SB	1219	1.8E−03	1.6E+05	2.1E−04	1.9E+04
*N. barri*	BT	3142	2.6E−03	2.3E+05	3.3E−04	2.9E+04
*P. hatchi*	ST	370	1.5E−03	1.4E+05	1.2E−04	1.0E+04
*P. hatchi*	GV	490	1.9E−03	1.7E+05	2.7E−04	2.4E+04
*P. hatchi*	SB	1219	2.2E−03	2.0E+05	3.3E−04	2.9E+04
*P. hatchi*	BT	3142	2.4E−03	2.2E+05	3.5E−04	3.1E+04

## Discussion

We used genome-wide genotyping to examine population structure of two troglobionts from the southern Cumberland Plateau in Tennessee. Despite relatively small distances between caves (no two caves were more than 15 km apart), we detected strong population structure for both species. Hierarchical clustering, PCA, and Admixture clearly identified each cave as a genetically distinct population (Figs. 2, 3). Pairwise estimates of genetic differentiation further supported these results, with a minimum weighted F_ST_ of 0.33 for *N. barri* and 0.30 for *P. hatchi* (Table 2), indicating “very great differentiation”^50^. For comparison, a recent 2bRAD study on the coral *Acropora millepora* across the Great Barrier Reef, including sites located over 1200 km apart, detected a maximum pairwise F_ST_ of 0.014^27^. Kamimura et al.^51^ reported a similar comparison. Using 16 microsatellite markers, they compared populations of cave-dwelling barklice (genus *Neotrogla*) from two Brazilian caves less than 1 km apart. While the number of individuals was small (3 individuals from one cave and 8 from another) they reported significant genetic differentiation, with F_ST_ = 0.043. They contrasted this with a similar F_ST_ value of 0.042, for populations of *Drosophila americana* located more than 200 km apart^52^. While it is difficult to directly compare F_ST_ between different types of markers, such as SNPs and microsatellites, these comparisons highlight the relatively strong genetic isolation between populations of terrestrial cave-dwelling species. The tendency of cave populations toward greater genetic differentiation was also shown in the Mexican blind cavefish complex (*Astyanax mexicanus*). Here, using 26 microsatellite loci, applied to 11 cave and 10 surface populations, Bradic et al.^53^ detected generally greater differentiation between cave populations than surface populations, with F_ST_ ranging from 0.2 to 0.5 between cave populations and a maximum of 0.09 for surface populations. These findings extend the idea, even among aquatic vertebrates, that cave populations exchange migrants much less readily than surface populations. These diverse cases reiterate the historical understanding that highly localized distributions of subterranean organisms are shaped by limited dispersal capabilities^54^.

Based on hierarchical clustering and admixture analyses (Figs. 2, 3), we expected to find greater genetic differentiation between caves located in different watersheds. This pattern was consistent for *N. barri*, but not for *P. hatchi* (Fig. 4). Although all the several genetic difference estimates were tightly correlated (Fig. S1), only absolute genetic distance (d_XY_ from hard genotype calls) was consistently lower within watersheds for *P. hatchi*.

Based on π estimates, the effective population sizes of both species were surprisingly large (Table 3), especially as caves are expected to harbor relatively small populations^2^. Because Next-Generation sequencing reads originate from single molecules, they are subject to several sources of error such as DNA damage, PCR errors, and sequencing errors, which can inflate estimates of nucleotide diversity^55^. De novo locus generation likely further contributes to this bias. Fortunately, these error sources can be reasonably expected to influence all samples similarly^22^. Hence, while we believe absolute estimates of Ne reported here are likely inflated, and should be considered cautiously, relative comparisons of diversity are still reliable. This idea is illustrated by the concurrent associations between cave length and π estimated using Angsd and from hard genotypes, despite the roughly eightfold difference between them in absolute terms (Table 3; Fig. 5; Figure S2).

Based on our results, we conclude that gene flow between caves is rare. This is consistent with the inability of terrestrial troglobionts to traverse even small distances between caves (Fig. 1). Hence the analogy of caves to islands in a sea of surface habitat holds for these species^2,12^. It is thought that migration of troglobionts must occur via subterranean connections^2,5^. One possibility for such connections would be Mesovoid Shallow Substratum (MSS). These intermediate habitats can form as crevices beneath streambeds of temporary watercourses (alluvial MSS) and can harbor both epigean and subterranean fauna^23^. Consistent with this concept, hierarchical clustering of populations for both species paired caves by watershed, rather than physical distance (Figs. 1, 2). This possibly reflects greater frequency of rare subterranean connections, or more recent variance, between caves located in the same watershed. While the ranges of both our study species are limited, they still encompass dozens of cave populations across areas substantially larger than our study area^12,19^. These numerous populations, coupled with the extreme genetic differentiation observed here, suggests that extremely rare migration into an unoccupied cave can establish a population that subsequently becomes genetically distinct. Rare migrants may still occasionally reach the cave, but at such a low frequency that the population remains genetically distinct from other caves just one or a few km distant.

For both species, we detected a positive association between nucleotide diversity and cave length (Fig. 5; Fig. S2). While the number of caves in our study was too small for confident statistical analysis, the strength of the correlation, and the similar patterns observed for the two different species, is an interesting trend consistent with the intuitive idea that larger caves harbor larger, more genetically diverse populations. Souza-Silva et al.^56^ reported a similar pattern, linking caves’ invertebrate species richness with their linear development. One explanation for this pattern is greater availability of food, and/or microhabitats in longer caves^56^. For instance, larger caves are known to harbor richer and more abundant bat populations^57^, that would provide larger guano deposits^56^. Another interesting possibility is that linear development increases caves’ exposure to small-scale interconnections, such as canaliculi^56^, or other intermediate subterranean environments^5,23,24^. This scenario would be particularly powerful in shaping richness if individuals in these intermediate connections were attracted into the larger caves by food sources such as guano^56^. At the regional scale, the number of caves^14,58^, rather than total karst area, is a significant predictor of troglobiont richness, indicating that while intermediate subterranean environments may be important for connectivity, caves are still a dominant factor shaping community diversity. The positive trend identified here (Fig. 5) is suggestive that similar processes may shape genetic diversity of individual troglobiont populations. Further application of genomic tools, as applied for the first time to the species in this study, will help shed light on how generally this pattern occurs.

## Supplementary information

Supplementary Figures.

## Data Availability

Demultiplexed reads for all samples are available on the NCBI SRA database (PRJNA601737). All scripts used for data processing, statistical analysis, and plotting figures, as well as intermediate data files, are available on github: https://github.com/grovesdixon/caveRAD. The permanent release of this repository linked to this publication is available here https://doi.org/10.5281/zenodo.4034777.
