# Supplementary material for: Population genomics in two cave-obligate invertebrates confirms extremely limited dispersal between caves
Source: Sci Rep. 2020 Oct 16;10:17554. doi: 10.1038/s41598-020-74508-9 (PMC7568537; doi:10.1038/s41598-020-74508-9)
Supplement: Supplementary file 1 — Supplementary Figures. [file 41598_2020_74508_MOESM1_ESM.pdf]

Supplementary Figures

Manuscript title:

Population genomics in two cave-obligate invertebrates confirms extremely limited dispersal between caves

Author names and affiliations:

Andras Balogh[1],

Lam Ngo[2],

Kirk S. Zigler[2],

Groves Dixon[1,*]

[1]Department of Integrative Biology, University of Texas, Austin, TX, USA

[2]Department of Biology, University of the South, Sewanee, TN, USA

*Corresponding author:

Groves Dixon

PAT Building room 427

2401 Speedway

grovesdixon@gmail.com

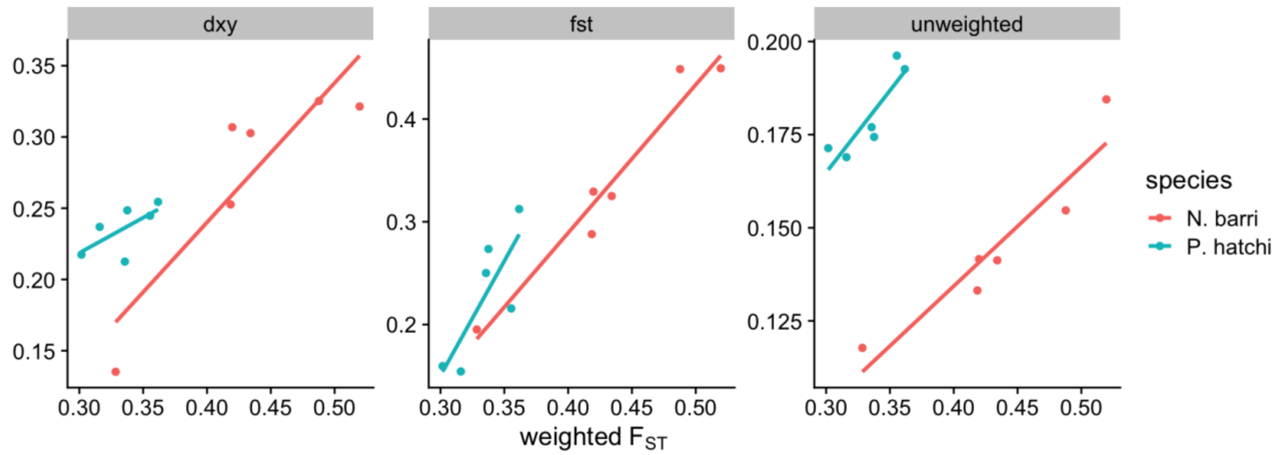

Figure S1: Correlation between pairwise estimates of genetic differentiation. The X axes show weighted F_{ST} computed using Angsd. The Y axes show the statistic indicated in the panel title: dxy = absolute genetic distance averaged across all variant sites from hard genotype calls; fst = Weir and Cockerham's F_{ST} averaged across all variant sites from hard genotype calls; unweighted = unweighted F_{ST} computed using Angsd.

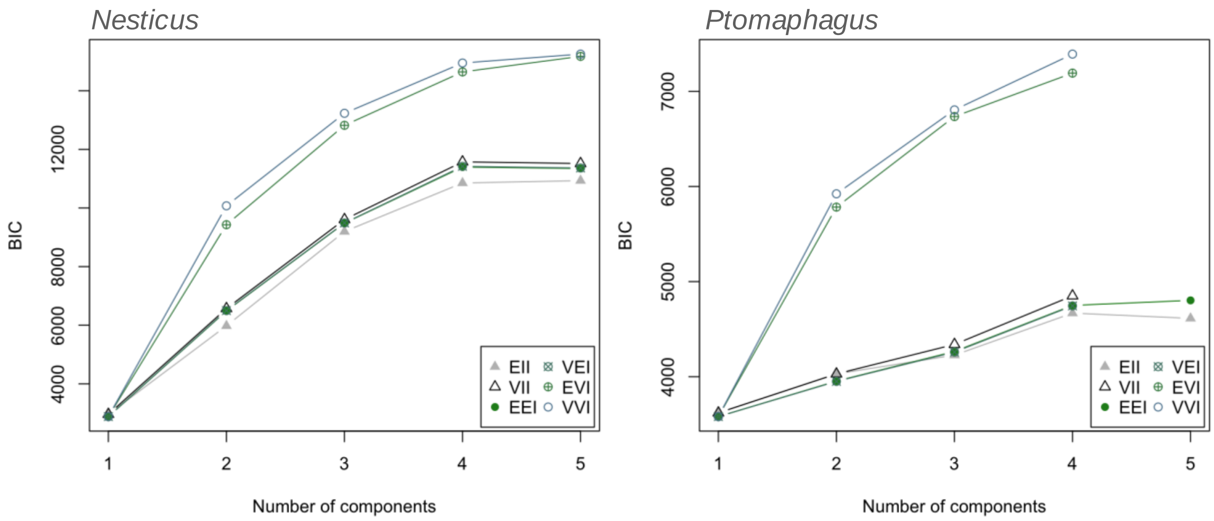

Figure S2: BIC values for clustering of samples. Depending on the model used, the optimal number of clusters was either 4 or 5 for each species. For *Nesticus*, 5 clusters had only marginally higher BIC than 4. Model abbreviations: EII: spherical, equal volume; VII: spherical, unequal volume; EEI: diagonal, equal volume and shape*; VEI:* diagonal, varying volume, equal shape*; EVI:* diagonal, equal volume, varying shape; VVI: diagonal, varying volume and shape. Unplotted components indicate failures of the model to converge.

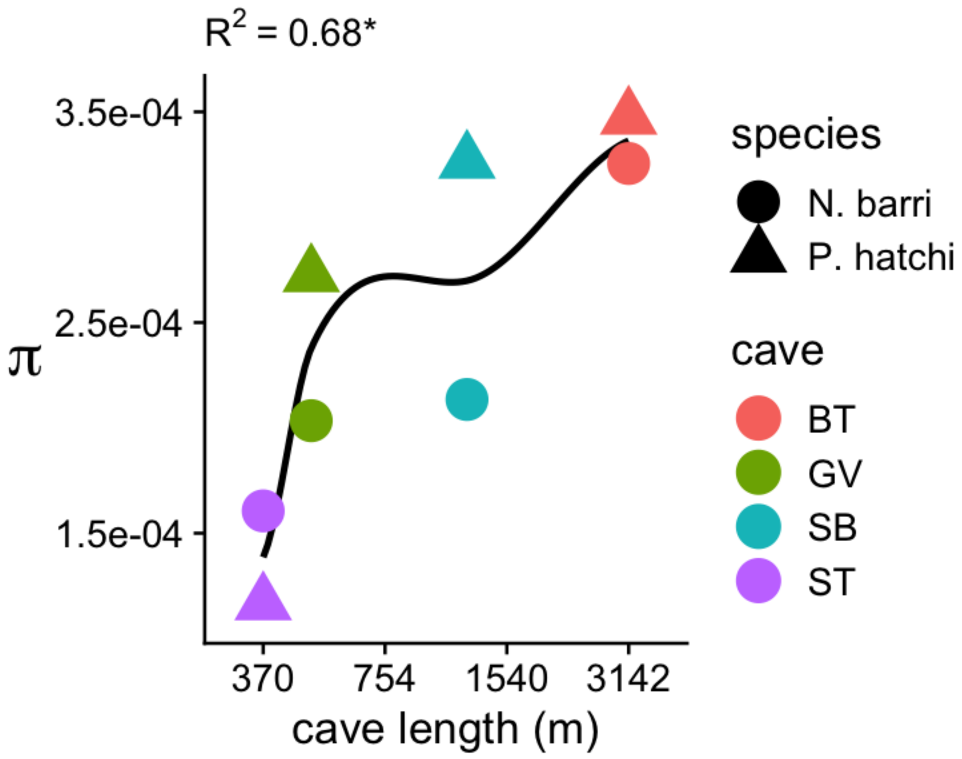

Figure S3: Relationship between per site nucleotide diversity (π) and cave length. X axis cave length on the log scale. Y axis shows the nucleotide diversities for each species caves pair computed from hard genotype calls. Point color indicates cave and point shape indicates species. Black line traces a locally smoothed regression for all points. R^2 for the linear model for all points is given above the plot ($p < 0.01$).
